# Joint reconstruction of quantitative T_2_ and apparent diffusion coefficient (ADC) maps in the heart

**DOI:** 10.1186/1532-429X-17-S1-W19

**Published:** 2015-02-03

**Authors:** Eric Aliotta, Daniel B Ennis

**Affiliations:** 1 Radiology, UCLA, Los Angeles, CA, USA; 2 Biomedical Physics IDP, UCLA, Los Angeles, CA, USA

## Background

Myocardial tissue characterization with T_2_-weighted imaging is an established technique for evaluating the presence of myocardial edema or iron overload (Kellman, P. *MRM* 2007, Anderson, LJ. *EHJ* 2001). More recently, both T_2_-mapping and apparent diffusion coefficient (ADC) mapping have emerged as quantitative techniques for characterizing edema (or iron overload) and water mobility. The purpose of this work was to develop a framework for the simultaneous recovery of both T_2_ and ADC from a single breath-hold acquisition.

## Methods

Spin echo (SE) diffusion weighted imaging (DWI) signals are principally governed by the tissue's apparent diffusion coefficient (ADC=D) and T_2_ relaxation, as well as the sequence's diffusion encoding b-value (b) and echo time (TE): S(b,TE) = S_0_e^-bD^e^-TE/T2^. We propose that acquisition of several signals with varying TEs and b-values permits joint reconstruction of both ADC and T_2_ maps.

Bloch equation simulations were used to generate signals for a broad range of T_2_ (20-70ms) and ADC (0.1-2.4x10^-3^mm^2^/s) using 10 TEs (17-100 ms) and b=500 s/mm^2^ (TE=60-68ms) along 3 directions. Complex Gaussian noise was added to each signal such that the signal to noise ratio (SNR) of the minimum TE, b=0 signal matched that of acquired data (SNR = 38). Reconstructions were performed using linear least-squares on a subset of the simulated data (TE=17,20,30,50,70,100ms) to reflect a feasible *in vivo* acquisition (scan time:18s). Mapping accuracy and precision were determined by the bias and standard deviation (SD) of T_2_ and ADC compared to programmed values.

Images were acquired on a 3.0 T Siemens Skyra system in an ex vivo infarcted porcine heart using single-shot SE EPI with TEs and b-values to match simulated parameters. T_2_ and ADC maps were jointly reconstructed using linear least-squares from 6 TEs plus 3 DWI sets and compared to: 1) Best-Available T_2_-maps from all 10 TEs; 2) Best-Available ADC maps from DWI (3 directions, 6 averages); 3) Independent T_2_ maps from 6 TEs; and 4) Independent ADC maps from 3 DWI averages.

## Results

Joint reconstruction of simulated data recovered T_2_ and ADC values with bias<1% and SD<10% for a broad range of tissues and even lower for healthy and infarcted myocardium (Table 1).

**Table 1:** Simulation results

	T2 Bias	T2 SD	ADC Bias	ADC SD
Healthy (T2=56ms, ADC=1.69x10-3mm2/s)	0.3 %	4.5 %	-0.3 %	7.0 %

Infarction (T2=69ms, ADC=2.4x10-3mm2/s)	0.2 %	4.6 %	0.2 %	5.6 %

**Figure 1:**
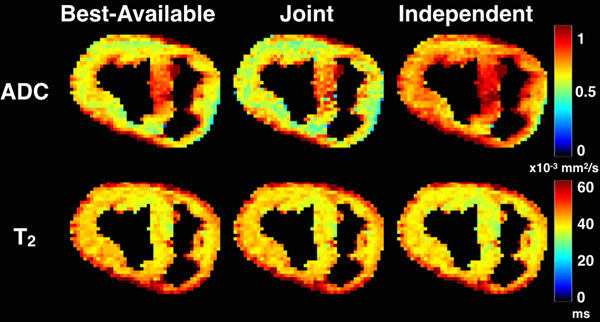
*Ex vivo* ADC and T_2_ maps from 10 TEs and 6 DWI averages, respectively (left), 6 TEs and 3 DWI averages (right) and Joint reconstruction from 6 TEs and 3 DWI sets (center). Joint estimation maps were closer to the Best-Available T2 or ADC maps than the Independent T2 or ADC maps alone and came with no increase in scan time.

Reconstructed ADC and T_2_ maps from the *ex vivo* acquisition are shown in Figure 1. Joint estimation maps were closer to the Best-Available T_2_ or ADC maps than the Independent T_2_ or ADC maps alone (Joint Estimation Maps: T_2_-bias=-0.5 %, ADC-bais=-4.8%; Independent Maps: T_2_-bias=-4.1%, ADC-bias=-14.1%).

## Conclusions

Joint acquisition and estimation of T2 and ADC maps is feasible in a breath hold and improves quantitative accuracy and precision compared to independent T2 or ADC mapping. DWI acquisitions typically require multiple averages to improve SNR. Here, varying TE takes the place of signal averaging and permits the reconstruction of a perfectly registered T_2_ map.

## Funding

This research was supported by Siemens Medical Solutions and the Department of Radiological Sciences at UCLA.

